# Resting-state functional connectivity and cognitive impairment after COVID-19 infection: Evidence from a large-scale fMRI study

**DOI:** 10.1192/j.eurpsy.2026.12227

**Published:** 2026-06-08

**Authors:** Andrea Perrottelli, Silvana Galderisi, Mario Amore, Stefano Barlati, Giammarco Cascino, Mario Cirillo, Daniele Corbo, Giorgio Di Lorenzo, Francesco Di Salle, Fabrizio Esposito, Roberto Gasparotti, Luigi Giuliani, Palmiero Monteleone, Gabriele Nibbio, Lorenzo Onorato, Antonio Vita, Mario Maj

**Affiliations:** 1 University of Campania “Luigi Vanvitelli”, Naples, Italy; 2 Department of Neuroscience, Rehabilitation, Ophthalmology, Genetics, Maternal and Child Health (DINOGMI), Section of Psychiatry, University of Genoa, Genoa, Italy; 3 IRCCS Ospedale Policlinico San Martino, Genoa, Italy; 4 Department of Clinical and Experimental Sciences, University of Brescia, Brescia, Italy; 5 Department of Medicine, Surgery and Dentistry “Scuola Medica Salernitana”, University of Salerno, Salerno, Italy; 6 Department of Medical and Surgical Specialties, Radiological Sciences and Public Health, University of Brescia, Brescia, Italy; 7 Department of Systems Medicine, University of Rome Tor Vergata, Rome, Italy

**Keywords:** cognitive impairment, COVID-19, fMRI, functional connectivity, SARS-CoV-2

## Abstract

**Background:**

The development of cognitive impairment (CI) is a frequent and debilitating consequence of COVID-19 and can persist for more than 1 year after the acute infection stage. Previous neuroimaging studies in COVID-19 survivors with CI have revealed widespread alterations in functional connectivity (FC), particularly within fronto-parietal circuits and subcortical nuclei such as the hippocampus, basal ganglia, and thalamus. This study focuses on neural correlates of CI in subjects who recovered from COVID-19 and the relationship between FC patterns and discrete cognitive domains.

**Methods:**

Resting-state functional MRI data from 136 subjects were analyzed using a ROI-to-ROI approach across 246 brain regions derived from the Human Brainnetome Atlas. Group comparisons were performed based on the presence or absence of CI, and correlation analyses were conducted between FC and scores obtained from a comprehensive neuropsychological test battery.

**Results:**

Whole-brain FC showed no difference between patients with and without CI. In the whole study sample, hypoconnectivity between two basal ganglia regions and two frontal motor regions was associated with impaired performance in the cognitive domain “Reasoning and Problem-Solving,” while hyperconnectivity between the prefrontal thalamus and the postcentral gyrus was associated with impaired performance in the same cognitive domain.

**Conclusions:**

Our findings indicate that FC alterations within the cortico-striatal and thalamo-cortical circuits may subtend deficits in higher-order executive functions in post-COVID-19 patients and highlight the importance of examining discrete cognitive domains in relation to brain connectivity.

## Background

More than one-third of patients who recovered from COVID-19 present a cognitive impairment (CI), with a prevalence ranging from 43.0 to 66.8% in severe cases [1–5]. CI is regarded as one of the most burdensome long-term consequences among COVID-19 survivors and, as observed in other psychiatric disorders, it has a strong impact on patients’ global functioning and quality of life [6–12]. We reported a frequency of CI of 35% in a large sample of patients who recovered from severe COVID-19 [13]. In line with other studies, we found that affected cognitive domains include executive functions, visual and verbal learning, attention, working and episodic memory, problem solving, and processing speed [5, 13, 14].

Although elucidating the neural processes underlying CI is of critical importance for developing effective treatment strategies, the biological underpinnings of post-COVID CI remain unclear [15]. Resting-state functional magnetic resonance imaging (rs-fMRI) enables the assessment of brain activity during rest and has been widely employed to investigate cerebral dynamics [16, 17]. Specifically, in rs-fMRI, functional connectivity (FC) refers to interregional synchrony, which is typically quantified by correlating low-frequency blood oxygen level-dependent (BOLD) fluctuations between predefined regions of interest (ROIs) or networks [16–18]. The underlying assumption is that voxels with similar BOLD time courses are functionally connected, reflecting coordinated activity within distributed brain networks supporting cognitive functioning [18]. On this basis, rs-fMRI provides a tool not only to characterize intrinsic neural networks supporting cognitive functioning but also to investigate how disruptions in these networks contribute to CI. Accordingly, meta-analyses have demonstrated associations between FC alterations and CI across multiple clinical conditions [19–21]. For instance, in mild cognitive impairment and Alzheimer’s disease, reduced connectivity within core hubs of the default mode network (DMN) (e.g., posterior cingulate cortex, precuneus) has been linked to deficits in memory and executive functions [19, 20]. Furthermore, in subjects with schizophrenia, several studies have shown that alterations in FC correlate with impairments in working memory, executive functions, and processing speed [22, 23]. Finally, FC measures have been shown to predict individual differences in cognitive performance across aging and clinical cohorts, further supporting their utility as potential biomarkers of CI [24–26].

Therefore, neuroimaging research may also contribute to the identification of neurobiological pathways underlying CI following SARS-CoV-2 infection. In fact, several rs-fMRI studies have detected alterations in FC in individuals who experienced COVID-19. In patients with post-COVID syndrome, a whole-brain FC analysis revealed that impaired memory performance was associated with reduced interhemispheric connectivity in the parahippocampal gyri, and decreased connectivity between bilateral orbitofrontal cortices and the cerebellar vermis [27]. In a larger patient sample, the same research group reported that attention and executive function scores were associated with alterations of the FC between the orbitofrontal cortex and the left superior motor area, cingulate cortex, and cerebellum [28]. Another study, conducted between 6 and 9 months after COVID-19 infection, found that an impairment in executive functions was linked to abnormal connectivity between the prefrontal cortex, superior parietal lobule, and somatosensory cortex, while impaired episodic memory performance was associated with hypoconnectivity between the hippocampus, on the one side, and the amygdala, cerebellum, subcortical structures (e.g., nucleus accumbens, subthalamic nuclei), and the orbitofrontal cortex, on the other side [29].

Other investigations adopted region-of-interest (ROI) and seed-based analytical frameworks. Dacosta-Aguayo et al. focused on the bilateral hippocampi of long-COVID patients assessed approximately 2 years after the infection, and found that a more severe memory impairment was associated with an increased connectivity of the hippocampus with multiple cortical and subcortical regions [30]. Moreover, a seed-to-voxel study revealed FC alterations between the thalamus and the primary motor cortex (M1), supplementary motor area (SMA), and anterior cingulate cortex (ACC); the hypoconnectivity between these regions correlated with reduced processing speed [31].

Studies employing independent component analysis (ICA) also highlighted FC disruptions within large-scale networks. In a study comparing cognitively impaired (COG+) and unimpaired (COG–) individuals after COVID-19 infection, tensorial ICA (a multivariate ICA approach that jointly decomposes spatial and temporal dimensions of fMRI data) [32] revealed increased connectivity in the right middle frontal gyrus (a region that links the ventral and dorsal attention networks) and reduced connectivity in the right inferior parietal lobule and left frontoparietal junction in the COG+ group [33]. Analyzing patients during the acute phase of the illness, Sarvandani and colleagues, employing the ICA approach, found that global cognitive scores, assessed by the Montreal Cognitive Assessment (MoCA), were associated with increased connectivity within the DMN, Dorsal Attention Network (DAN), and with decreased connectivity within networks involving the primary visual and frontal cortices [34]. Interestingly, a recent study reported that subjects with post-COVID-19 syndrome showed increased intra-network FC across 10 cerebral networks, particularly those implicated in cognition, including the DMN, salience, executive control, auditory, and basal ganglia networks, compared to healthy controls [35]. However, no significant differences in FC, either within or between networks, were observed when comparing subjects with CI to those without CI. Finally, the same study by Dacosta-Aguayo, previously mentioned for its hippocampal ROI-based connectivity findings, also employed an ICA approach in the same cohort and reported an association between impaired memory performance and higher resting-state activity in the right frontoparietal network [30].

Overall, cognitive deficits appear to involve both hypo- and hyper-connectivity across multiple brain regions, reflecting the multifaceted nature of COVID-19–related cognitive dysfunction. However, several studies are limited by methodological constraints, including modest sample sizes, use of different measures of cognitive performance, MRI analysis limited to a few cerebral regions based on a priori hypotheses, and variability in study objectives. These limitations may hinder comparability among studies and contribute to inconsistent findings.

To address these limitations, the present study was designed to: (1) assess CI by means of a screening neurocognitive test administered face-to-face to a large sample of patients who had been either hospitalized for symptomatic COVID-19 infection, or had not been hospitalized but experienced fever for at least 5 days or a body temperature higher than 38.0 °C for at least 3 days after confirmed COVID-19 infection; (2) collect rs-fMRI data to identify FC patterns associated with global CI, as measured by the Montreal Cognitive Assessment (MoCA); (3) evaluate the relationships between specific alterations in rs-fMRI derived FC and impairment in discrete cognitive domains, as measured through a comprehensive neuropsychological battery.

Given the previous findings in COVID-19 survivors, we hypothesize that subjects with CI will show FC alterations primarily among regions of the DMN, DAN, and visual and saliency networks, including fronto-parietal areas, limbic and medial temporal regions, and subcortical nuclei [36]. However, we also predict that the heterogeneity in the clinical presentation of post-COVID-19 syndrome and its diverse impact on cognitive domains may limit the ability to draw robust conclusions regarding the relationship between FC alterations and CI across our entire cohort. In addition, given the extensive evidence that CI is indexed by FC across distributed brain areas, we expect to observe significant correlations between FC measures of cortical–subcortical loops and assessment of cognitive functioning [34, 35, 37].

## Methods

### Participants

Participants in the clinical-neurocognitive study were recruited at the infectious disease units of five Italian University Hospitals (University of Brescia, University of Campania Luigi Vanvitelli, University of Genoa, University of Rome “Tor Vergata,” and University of Salerno). The enrollment process started in March 2021 and ended in September 2022. Inclusion criteria were: (a) age between 18 and 65 years; (b) a history of confirmed SARS-CoV-2 infection (i.e., positive RT-PCR test); (c) previous hospitalization due to COVID-19 disease or, if not hospitalized, confirmed COVID-19 infection with concomitant symptoms including either fever for at least 5 days or body temperature higher than 38 °C for at least 3 days; (d) being followed on an outpatient basis by one of the infectious disease units of the study centers; (e) absence of SARS-CoV-2 infection at the time of study recruitment; (f) willingness to sign the informed consent. Exclusion criteria were: (a) history of CI prior to COVID-19 infection, and (b) history of chronic psychosis, bipolar disorder, major depression, neurological disease, or head injury prior to COVID-19 infection. The study was approved by the Ethics Committees of participating centers and was conducted in accordance with the principles of the Declaration of Helsinki (59th World Medical Association General Assembly; October 2008).

A comprehensive written explanation of the study procedures and objectives and a written informed consent form relevant to the participation in the study and personal data processing were provided to all subjects who met the inclusion/exclusion criteria reported above, as verified by two ad hoc forms. Demographic data (sex, age, education) and the severity of COVID-19 disease at the time of symptomatic SARS-CoV-2 infection were recorded on a 1 to 4 scale (mild to critical) defined according to the US Centers for Disease Control and Prevention criteria [38].

### Cognitive assessment

#### Screening and baseline assessment measures

During the screening phase, the MoCA was used to evaluate executive functions, working memory, attention, language, abstract reasoning, visuospatial ability, delayed recall, and orientation [39]. A total score below 26 indicates CI, with a one-point adjustment for subjects with less than 12 years of education.

The Measurement and Treatment Research to Improve Cognition in Schizophrenia Consensus Cognitive Battery (MCCB) was used at the baseline phase of the study to obtain a comprehensive assessment of cognition. It includes 10 tests that investigate seven cognitive domains (speed of processing, attention/vigilance, working memory, verbal learning, visual learning, reasoning and problem-solving, and social cognition) [40, 41]. Raw scores were converted to standardized T-scores and were corrected for age, gender, and education using the Italian normative data. The Neurocognitive Composite T-score (using the six neurocognitive domains) and the Overall Composite T-Score (using the six neurocognitive domains and the social cognition domain) were also obtained.

The Hamilton Depression Rating Scale 17-item (HAM-D-17) was used to evaluate the severity of depressive symptoms for the week preceding the assessment [42]. It is a 17-item scale (the higher the score, the more severe the depressive symptoms).

The Hamilton Rating Scale for Anxiety (HAM-A) consists of 14 items and was used to assess the severity of both psychic and somatic anxiety symptoms [43].

The Impact of Event Scale-Revised (IES-R) [44] is a questionnaire measuring current subjective distress in response to a specific traumatic event. It includes 22 items, each rated on a 0–4 scale. A total score of 24 to 32 indicates mild psychological impact, 33 to 36 moderate, and from 37 on a severe impact.

#### Statistical analyses on demographic, COVID-19 disease, and cognitive profile

Enrolled subjects were divided into two groups based on the presence (COG+) or absence (COG−) of cognitive impairment, as assessed by the MoCA. Continuous demographic variables were reported as mean ± standard deviation (SD), while categorical variables were reported as frequencies.

Comparisons between study groups on demographic variables, MoCA scores, and severity of COVID-19 during the acute phase were conducted using independent-samples *t*-tests and χ^2^ tests. In addition, a multivariate analysis of variance (MANOVA) was performed on MCCB composite scores and individual domain *T*-scores, followed by post hoc tests corrected for multiple comparisons (*p* < 0.0055) to compare COG+ and COG− subjects.

### Neuroimaging

#### MRI data acquisition

Subjects who completed the screening and baseline evaluations of the clinical study were invited to participate in the MRI add-on study. All participants underwent a structural MRI (sMRI) and an rs-fMRI acquisition. The detailed MRI scan protocols are provided in the Supplementary Material. For the present study, MRI data had to be acquired within 90 days of the baseline phase. Furthermore, participants from centers contributing fewer than 10 subjects were excluded from the analysis to reduce data heterogeneity due to differences in MRI scanners and site-specific factors.

#### Pre-processing

Functional and anatomical data were preprocessed using a standard preprocessing pipeline with the CONN functional connectivity toolbox [45] release 22.a and SPM12. The preprocessing pipeline included realignment with correction of susceptibility distortion interactions, slice timing correction, outlier detection, indirect segmentation and MNI-space normalization, and smoothing. Detailed information on fMRI data preprocessing is provided in the Supplementary Material.

#### ROI selection

For the extraction of signal time courses in each anatomical district, the Human Brainnetome Atlas (BNA) was employed [46]. The BNA divides the brain into 246 regions of interest (ROIs), 123 per hemisphere, comprising 210 cortical and 36 subcortical ROIs (Supplementary Figure S1). Due to the diverse results on the areas involved in post-COVID CI, ROIs were not selected based on a priori hypotheses; instead, a whole-brain approach was used, including all atlas-defined regions to comprehensively characterize all the possible patterns of FC alterations associated with cognitive functioning in this dataset.

#### First-level analysis

First-level analysis: ROI-to-ROI connectivity (RRC) matrices were estimated, characterizing the FC between each pair of regions among 246 ROIs. Functional connectivity strength was represented by Fisher-transformed bivariate correlation coefficients from a general linear model [weighted-GLM, [47]] estimated separately for each pair of ROIs, characterizing the association between their BOLD signal timeseries.

#### Group-level analysis

Group-level analyses were performed using a General Linear Model (GLM) [47]. For each individual connection a separate GLM was estimated, with first-level connectivity measures at this connection as dependent variables (one independent sample per subject), and groups (COG+ and COG−) as the independent variable. Participants’ age, sex, and site of MRI acquisition (center) and other potential confounding variables resulting from additional analyses were included as covariates during second-level group analysis. Methodological details regarding the connection level hypothesis, clustering procedure, and connectivity metrics for this analysis are provided in the Supplementary Material.

Group-level results were thresholded using a combination of a *p* < 0.05 connection-level threshold and a familywise corrected *p*-FDR < 0.05 cluster-level threshold [48].

#### Correlations between functional connectivity and cognitive variables

Second-level ROI-to-ROI analyses were performed using regression models within a GLM framework (connection-level threshold *p* < 0.05 and cluster-level FDR-corrected *p* < 0.05) to assess associations between functional connectivity and cognitive measures (scores recorded for MoCA, MCCB composite indices, and individual cognitive domains). Regression models were conducted both on the entire study sample and separately within the two groups (COG+ and COG−).

For each significant regression analysis indicating an association between one connectivity cluster and scores of a cognitive domain, the connectivity values (Fisher *z*-transformed correlation coefficients) of the constituent connections of the cluster were extracted. These values were then correlated with cognitive performance scores to further characterize the relationship between individual ROI-to-ROI connections and the cognitive index of interest. Normality tests were performed on FC data and on cognitive variables to assess the distribution of the data. Based on the results of normality tests, Pearson’s correlation coefficients (*r*) or Spearman’s rank correlations were performed using SPSS v.29 (IBM). Bonferroni correction was applied to control for type-I error inflation. Furthermore, for correlations with a significant *p*-value, partial correlations were performed to exclude the influence of psychopathological variables (anxiety, depression, and subjective distress in response to a specific traumatic event) and time between COVID-19 infection and screening.

## Results

### Study sample

**Figure 1.**
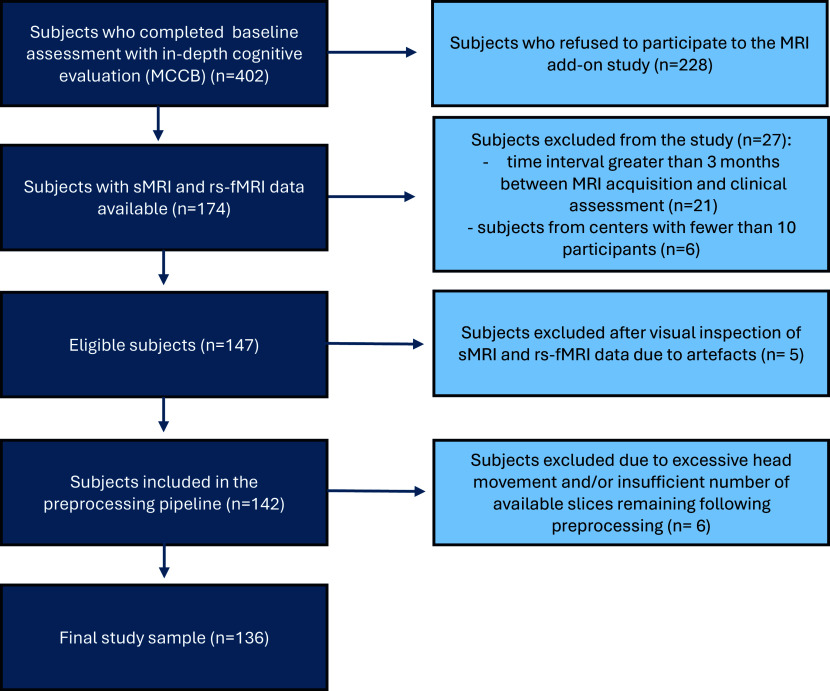
Included and excluded subjects. Figure 1. long description.

One hundred and seventy-four subjects from the clinical/neurocognitive study agreed to complete sMRI and rs-fMRI scans (Figure 1). After excluding 38 subjects (please refer to Figure 1 for details), the final sample included 136 subjects (Mean ± S.D. of days between baseline assessment and MRI acquisition = 52.2 ± 33.9 days). Based on the MoCA results, the COG+ group included 52 subjects (38.2%), and the COG- group 84 (61.8%).

### Demographic and cognitive profiles of the study sample

Differences in demographic characteristics between COG+ and COG− are reported in Table 1. COG+ subjects were significantly older and had lower scores on the MoCA, as compared to COG- subjects (*p* < 0.001).

**Table 1.** Characterization and comparison of the study groups based on the presence or absence of cognitive impairment (*n* = 136) Table 1. long description.

	COG+ (*n* = 52)	COG- (*n* = 84)	*t*-*χ*^2^-F/*p*-value
Age^b^	57.30 ± 7.30	50.73 ± 10.26	**4.361/ <0.001** ^a^
Gender (M/F)^b^	37/15	46/38	3.629/ 0.057
Education (Years)^b^	12.44 ± 3.58	14.01 ± 3.85	2.375/ 0.019
MoCA total score^c^	23.15 ± 2.29	27.65 ± 1.35	**12.873/ <0.001** ^a^
COVID–19 disease severity^c^ (at the time of symptomatic infection)	2.14 ± 0.98	1.89 ± 1.01	1.333/ 0.185
Time from COVID–19 Infection to screening (months)^c^	14.40 ± 8.90	16.98 ± 8.90	1.595/0.133
MCCB speed of processing^d^	47.41 ± 11.41	52.08 ± 8.82	3.994/0.048
MCCB attention/vigilance^d^	44.26 ± 11.32	49.56 ± 10.49	6.326/0.013
MCCB working memory^d^	42.10 ± 12.78	49.94 ± 8.20	**15.189/<0.001** ^a^
MCCB verbal learning^d^	41.49 ± 11.50	50.66 ± 10.70	**22.371/<0.001** ^a^
MCCB visual learning^d^	38.90 ± 14.80	52.48 ± 9.73	**43.308/<0.001** ^a^
MCCB reasoning and problem-solving^d^	43.69 ± 9.46	51.34 ± 9.39	**14.214/<0.001** ^a^
MCCB social cognition^d^	51.27 ± 12.58	53.91 ± 10.03	2.373/0.126
MCCB neurocognitive composite score^d^	40.28 ± 13.62	51.79 ± 8.46	**30.222/<0.001** ^a^
MCCB overall composite score^d^	39.23 ± 12.76	51.10 ± 8.48	**24.699/<0.001** ^a^

*Note: COG+: subjects who presented a MoCA score < 26;* *COG-* *:* *subjects who scored ≥26 on the MoCA evaluation.*

a **Significant difference** **(** ***p*** **-value** **threshold adjusted for multiple tests):**

b *p* < 0.0125 for demographic variables;

c *p* < 0.0125 for MoCA score & COVID-19 disease variables;

d *p* < 0.0055 for MCCB variables.

The MANOVA showed a significant difference in the MCCB scores between the COG+ and COG− groups [Wilks’ Λ = 0.62, *F*_(9, 101)_ = 6.681, *p* < 0.001]. Post hoc comparisons showed that the COG+ subjects had significantly lower overall composite (neurocognition plus social cognition) and neurocognitive composite scores, as well as lower scores on working memory, verbal learning, visual learning, and reasoning and problem-solving (*p* < 0.01) (Table 1).

### Group comparison on resting-state functional connectivity

**Figure 2.**
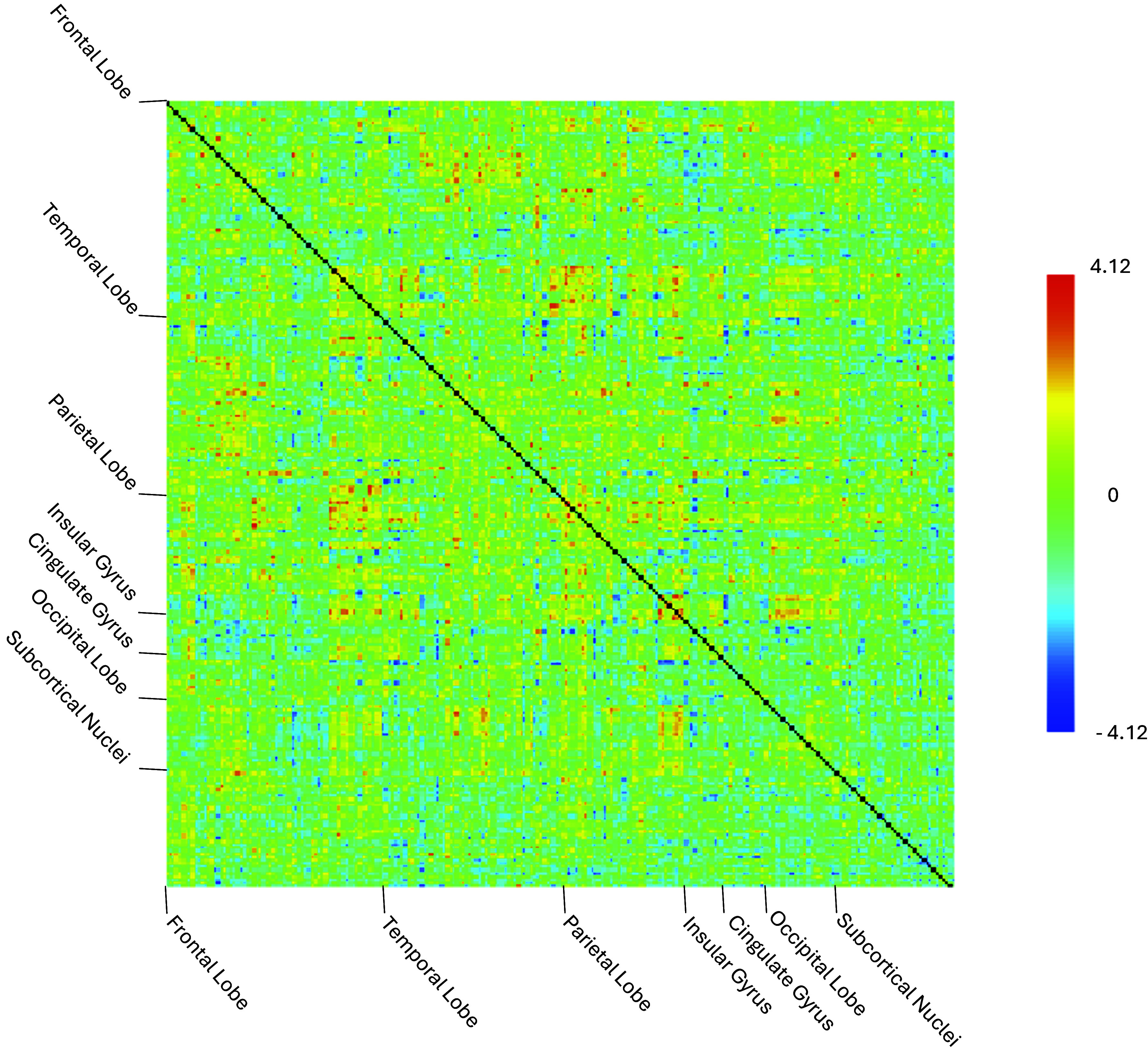
Unthresholded 246 X 246 ROI-to-ROI connectivity matrix obtained for the COG+ vs COG− contrast. *This matrix represents the full set of ROI* *-to* *-ROI* *associations prior to statistical thresholding or multiple comparison correction. No connections remained significant after applying the connection* *-level* *threshold* *(*p* < 0.05) and the cluster* *-level* *FDR correction* *(p-FDR < 0.05).* Figure 2. long description.

The second-level ROI-to-ROI analyses, performed across the 246 BNA ROIs to compare COG+ and COG– groups, yielded a single unthresholded 246 × 246 connectivity matrix (Figure 2), representing all pairwise Fisher-transformed correlations between ROIs. Group-level contrasts (COG+ > COG− and COG- > COG+) were then tested within a GLM framework. No significant between-group differences in FC emerged, either at the individual connection level or when applying cluster-level correction.

Controlling for age, sex, years of education, site, hospitalization due to COVID-19 disease, and time between COVID-19 infection and screening (entered individually and in combination as covariates in the GLM) did not modify the results.

### Correlations between resting-state connectivity and cognitive domains

**Figure 3.**
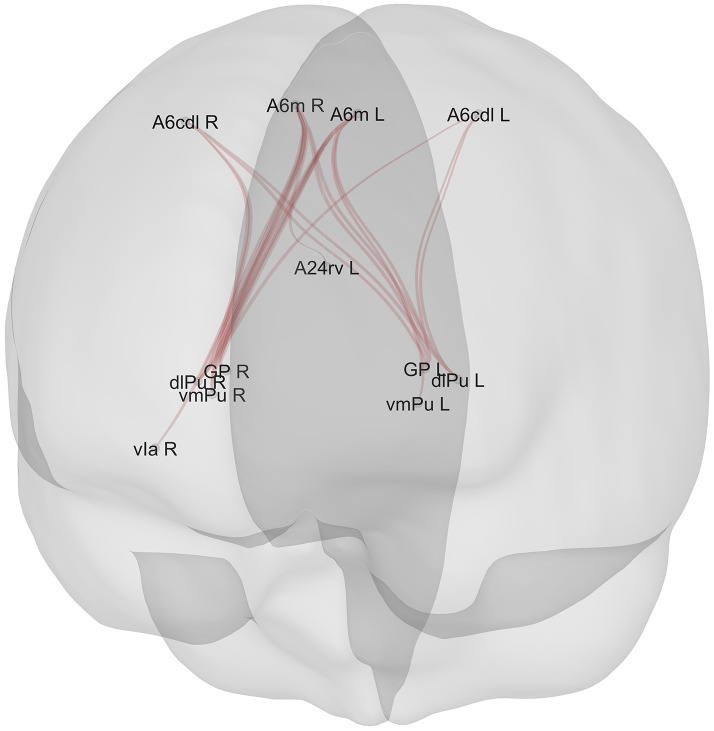
Positive associations (*n* = 20) between ROIs and scores on the reasoning and problem-solving domain in the whole sample (*n* = 136) (higher connectivity between these ROIs corresponded to better performance in the reasoning and problem-solving domain). *Legend: A6m_R: Superior Frontal Gyrus - Medial area 6* *– Right; A6m_L: Superior Frontal Gyrus – Medial area 6* *– Left; A6cdl_L: Precentral Gyrus – Caudal dorsolateral area 6* *– Left; A6cdl_R: Precentral Gyrus – Caudal dorsolateral area 6* *– Right; vIa_R: Insular gyrus, Ventral agranular insular – Right; vmPu_L: Ventromedial putamen – Left; dlPu_L: Dorsolateral putamen – Left; GP_L: Globus pallidus – Left; vmPu_R: Ventromedial putamen – Right; dlPu_R: Dorsolateral putamen – R; GP_R: Globus pallidus – Right; A24rv_L: Cingulate Gyrus, rostroventral area 24* *– Left.* ***N.B.*** *: The thickness of the lines does not represent the strength of connectivity; variations are due to the graphical perspective and overlap of multiple connections originating from the same* *ROI.* Figure 3. long description.

**Figure 4.**
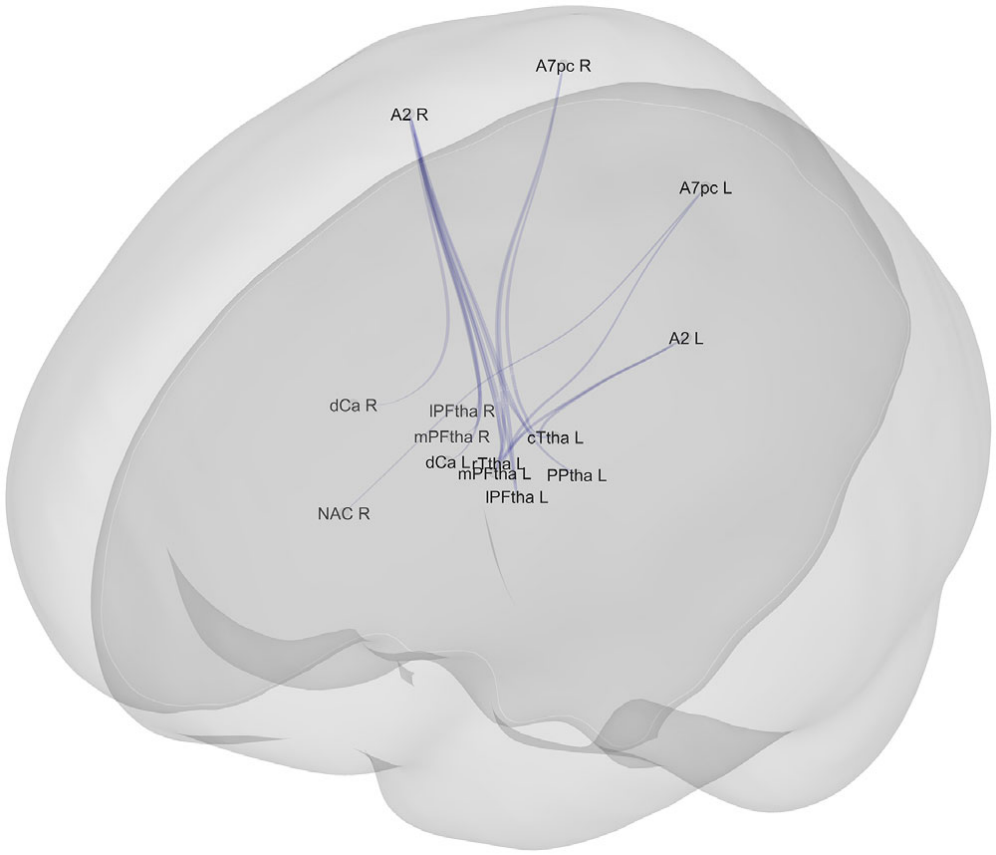
Negative associations (*n* = 17) between ROIs and scores of the reasoning and problem–solving domain in the whole sample (*n* = 136) (lower connectivity between these ROIs corresponded to worse performance in the reasoning and problem–solving domain). *Legend: A7pc_R: Superior Parietal Lobule, postcentral area 7* *– Right; A7pc_L: Superior Parietal Lobule, postcentral area 7* *– Left; A2_L: Postcentral Gyrus – Area 2* *– Left; A2_R: Postcentral Gyrus – Area 2* *– Right; cTtha_L: Caudal Temporal thalamus – Left; rTtha_L: Rostral Temporal thalamus – Left; dCa_L: Dorsal caudate – Left; dCa_R: Dorsal caudate – Right; NAC_R: Nucleus accumbens – Right; lPFtha_L: Lateral Prefrontal thalamus – Left; PPtha_L: Posterior parietal thalamus – Left; lPFtha_R: Lateral Prefrontal thalamus – Right; mPFtha_R: Medial Prefrontal thalamus – Right; mPFtha_L: Medial Prefrontal thalamus – Left.* *N.* *B.: the thickness of the lines does not represent the strength of connectivity.* ***N.B.:*** *the thickness of the lines does not represent the strength of connectivity; variations are due to the graphical perspective and overlap of multiple connections originating from the same* *ROI.* Figure 4. long description.

**Figure 5.**
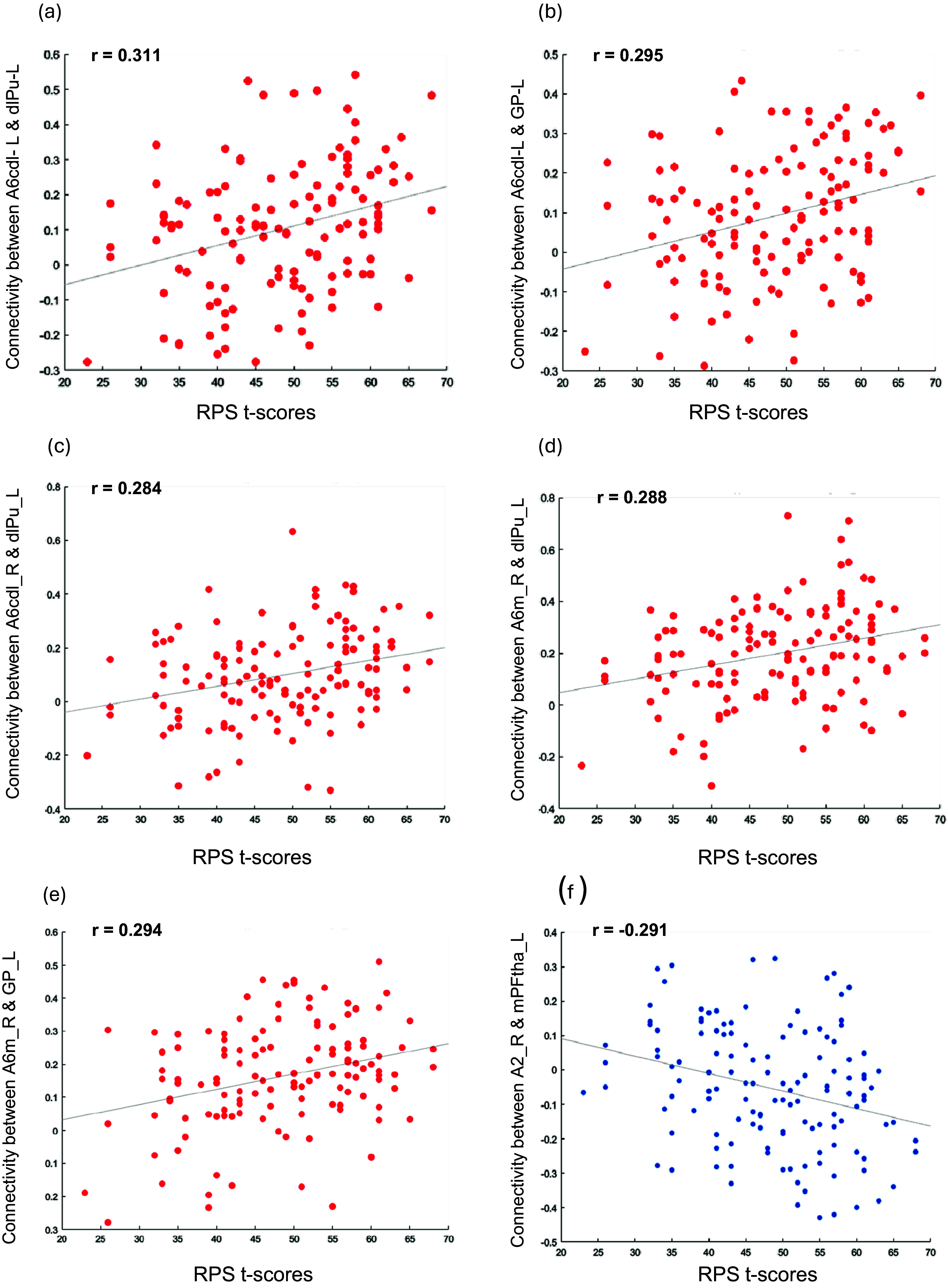
Scatter plot of the significant correlations between ROI-to-ROI connections and reasoning and problem-solving scores in the whole sample (*n* = 136). *The six scatter plots show the significant correlations [five positive* *(A–E) and one negative* *(F)] between reasoning and problem* *-solving* *scores and ROI* *-to* *-ROI* *connectivity indices between the:* *(A)* *Left Precentral Gyrus – Caudal dorsolateral area 6* *and Left Dorsolateral putamen;* *(B)* *Left Precentral Gyrus – Caudal dorsolateral area 6* *and Left Globus pallidus;* *(C)* *Right Precentral Gyrus – Caudal dorsolateral area 6* *and Left Dorsolateral Putamen;* *(D)* *Right Superior Frontal Gyrus – Medial area 6* *and Left Dorsolateral Putamen;* *(E)* *Right Superior Frontal Gyrus – Medial area 6* *and Left Globus pallidus;* *(F)* *Right Postcentral Gyrus – Area 2* *and Left Medial Prefrontal Thalamus.* Figure 5. long description.

FC showed no significant association with the MoCA and the two composite MCCB scores when considering the whole study sample (*n* = 136) or the COG+ and COG− groups separately, but was significantly associated with the reasoning and problem-solving scores in the whole study sample. Specifically, FC values of one cluster comprising 26 ROIs were significantly associated with reasoning and problem-solving performance (*F*_(3,127)_ = 9.39, *p*_uncorrected_ = 0.000012, *p*-FDR = 0.00554). Within this cluster, 20 positive (Figure 3) and 17 negative (Figure 4) correlations between ROI-to-ROI connectivity values and RPS scores were identified. Follow-up analyses showed that five of the 20 positive (Table 2) and one of the 17 negative (Table 3) correlations remained significant after correction for multiple tests. Impairments in the reasoning and problem-solving domain were associated with hypoconnectivity between the left dorsolateral putamen and three cerebral regions: the left and right precentral gyrus and the right superior frontal gyrus [Table 2; Figure 5(a–e)]; and between the left globus pallidus and two cerebral regions: the left precentral gyrus and the right superior frontal gyrus [Table 2; Figure 5(a–e)]. In addition, impairments in reasoning and problem-solving were associated with hyperconnectivity between the left medial prefrontal thalamus and the right postcentral gyrus [Table 3; Figure 5(f)].

**Table 2.** Positive correlations across the study sample (*n* = 136) between extracted connectivity values of ROI pairs of the significant cluster and reasoning and problem-solving scores Table 2. long description.

ROI *n.*1	ROI *n.*2	r	*p*-value
Precentral gyrus – caudal dorsolateral area 6 - L	Dorsolateral putamen – R	0.181	0.038855
	Dorsolateral putamen – L	0.311	**0.000294** ^a^
	Globus pallidus – L	0.295	**0.000632** ^a^
Precentral gyrus – caudal dorsolateral area 6 – R	Dorsolateral putamen – R	0.191	0.028831
	Dorsolateral putamen – L	0.284	**0.001016** ^a^
	Globus pallidus – L	0.226	0.009588
	Ventromedial putamen – R	0.212	0.014821
Superior frontal gyrus – medial area 6 – L	Dorsolateral putamen – R	0.197	0.023861
	Dorsolateral putamen – L	0.233	0.007284
	Globus pallidus – R	0.189	0.030368
Superior frontal gyrus – medial area 6 – L	Insular gyrus, Ventral agranular insular – R	0.193	0.026907
	Ventromedial putamen – R	0.199	0.022827
	Ventromedial putamen – L	0.246	0.004637
Superior frontal gyrus – medial area 6 – R	Cingulate Gyrus, rostroventral area 24	0.182	0.037254
	Dorsolateral putamen – R	0.197	0.023919
	Dorsolateral putamen – L	0.288	**0.000841** ^a^
	Globus pallidus – R	0.238	0.006144
	Globus pallidus – L	0.294	**0.00064** ^a^
	Insular gyrus, Ventral agranular insular – R	0.214	0.014083
	Ventromedial putamen – R	0.221	0.011274

a **Significant correlation** **(** ***p*** **-value** **threshold adjusted for multiple tests:** ***p*** **< 0.00135).**

**Table 3.** Negative correlations across the whole study sample (*n* = 136) between the extracted connectivity values of ROI pairs of the significant cluster and reasoning and problem-solving scores Table 3. long description.

ROI *n.*1	ROI *n.*2	r	*p*-value
Postcentral gyrus – area 2 - L	Caudal temporal thalamus – L	−0.210	0.0163
	Medial prefrontal thalamus – L	−0.220	0.0115
Postcentral gyrus – area 2 – R	Rostral temporal thalamus – L	−0.179	0.0411
	Caudal temporal thalamus – L	−0.271	0.0018
	Dorsal caudate – L	−0.204	0.0197
	Dorsal caudate – R	−0.181	0.0387
	Lateral prefrontal thalamus – L	−0.267	0.0021
	Lateral prefrontal thalamus – R	−0.247	0.0045
	Medial pre-frontal thalamus – L	−0.291	**0.000749** ^a^
	Medial prefrontal thalamus – R	−0.201	0.0213
	Posterior parietal thalamus – L	−0.263	0.0024
	Rostral temporal thalamus – L	−0.200	0.0222
Superior parietal lobule, postcentral area 7 – L	Nucleus accumbens – R	−0.191	0.0288
	Rostral temporal thalamus – L	−0.180	0.0402
Superior parietal lobule, postcentral area 7 – R	Caudal temporal thalamus – L	−0.175	0.0450
	Lateral prefrontal thalamus – L	−0.204	0.0192
	Medial prefrontal thalamus – L	−0.200	0.0223

a **Significant correlation** **(** ***p*** **-value** **threshold adjusted for multiple tests:** ***p*** **< 0.00135)**.

Finally, partial correlations indicated that five out of six correlations remained significant after controlling for anxiety, depression, and distress in response to traumatic events and the time between COVID-19 infection and screening (Table 4).

**Table 4.** Partial correlations across the whole study sample (*n* = 136) between the connectivity values of the ROI pairs of the significant cluster and reasoning and problem-solving scores Table 4. long description.

ROI *n.*1	ROI *n.*2	r	*p*-value
Precentral gyrus – caudal dorsolateral area 6 – L	Dorsolateral putamen – L	0.327	**0.000234** ^a^
	Globus pallidus – L	0.258	**0.00403** ^a^
Precentral gyrus – caudal dorsolateral area 6 – R	Dorsolateral putamen – L	0.292	**0.00106** ^a^
Superior frontal gyrus – medial area 6 – R	Dorsolateral putamen – L	0.259	**0.00394** ^a^
	Globus pallidus – L	0.227	0.0117
Postcentral gyrus – area 2 – R	Medial pre-frontal thalamus – L	−0.248	**0.00582** ^a^

a **Significant correlation** **(** ***p*** **-value** **threshold adjusted for multiple tests:** ***p*** **< 0.0083)** *and after correction for anxiety, depression, subjective distress in response to a specific traumatic event and time between COVID-19* *infection*.

## Discussion

The present study explores functional connectivity alterations in a large sample of patients after COVID-19 disease, focusing on neural correlates of cognitive deficits that develop and persist as a consequence of the infection. In line with a recent meta-analysis reporting CI in 33% of individuals assessed at least 1 year after the infection [14], in 136 post-COVID patients, we found that over one-third of them showed evidence of impaired visual learning, verbal learning, working memory, and reasoning, and problem-solving more than a year after the acute phase of the infection, a neurocognitive profile consistent with the “dysexecutive syndrome” in long COVID described in a recent review [49].

We found no significant group differences in FC between patients with and without CI, but found an association between FC values and performance in the reasoning and problem-solving domain, a key component of executive functions that specifically reflects problem-solving abilities. We identified two main hubs of connectivity associated with impairments in reasoning and problem-solving: (1) connections between divisions of the basal ganglia and the frontal lobe, and (2) connections between the thalamus and the postcentral gyrus. As for the first hub, the hypoconnectivity between two basal ganglia regions (the dorsolateral putamen and globus pallidus) and two frontal motor regions (the caudal dorsolateral division of the precentral gyrus and the medial division of the superior frontal gyrus) was associated with impairments in reasoning and problem-solving. The basal ganglia are crucial for decision-making and executive functions, and neuroimaging alterations in these regions have been consistently associated with CI in psychiatric and neurological conditions [50, 51]. Within the basal ganglia, the striatum, which includes the caudate nucleus, putamen, and nucleus accumbens, coordinates both motor and cognitive functions [52, 53]. Specifically, previous studies have shown that the putamen plays a key role in verbal learning, executive functioning, and working memory [54, 55]. In addition, within the basal ganglia-cortical circuitry, the globus pallidus is a structure that receives striatal outputs and has been implicated in motor planning and execution, and cognitive control [55–57]. In COVID-19 patients, studies have highlighted alterations in structural and functional MRI data related to the basal ganglia subdivisions, persisting even several months after the infection [29, 58–61]. One ROI-to-ROI fMRI study highlighted hyperconnectivity between the bilateral caudate nucleus and the left prefrontal cortex in subjects with severe COVID-19 during the acute phase, which persisted almost 1 year after infection [29]. Interestingly, and in line with our results, one study showed that hypoconnectivity within the basal ganglia network correlated with CI (MoCA total score) [35]. Finally, one study [62] investigated connectivity alterations between the basal ganglia and other brain regions, showing that individuals with long COVID exhibited increased connectivity between: (1) the right posterior caudate and bilateral precentral gyri and (2) the right anterior globus pallidus and both posterior cingulate cortex and the right temporal pole [62]. Moreover, hyperconnectivity between the right posterior caudate and the left precentral gyrus was associated with CI and deficits in cognitive flexibility. However, it is worth noting that the findings by Troll et al. [62] revealed a significant association with CI in the opposite direction from ours. However, they reported a link between the left precentral gyrus and the caudate nucleus, while we observed associations involving the connectivity of the precentral gyrus with the putamen, and, in addition, their study used different recruitment criteria and their assessments were conducted earlier post-infection (9 months on average).

Overall, available findings suggest a complex and potentially bidirectional relationship, encompassing both hypo- and hyper-connectivity, between corticostriatal circuits and CI in COVID-19.

The second main finding of our study was that hyperconnectivity between the prefrontal thalamus and the postcentral gyrus was significantly associated with greater impairments in the reasoning and problem-solving domain. The thalamus is a subcortical structure composed of multiple nuclei that act as a relay hub, integrating and transmitting sensory, motor, and cognitive information between cortical and subcortical regions [63]. The postcentral gyrus, located in the parietal lobe, comprises the primary somatosensory cortex and is responsible for processing tactile and nociceptive input from the body [64]. Together, these regions contribute to sensory perception, sensorimotor integration, and higher-order cognitive functions [63, 65]. Alterations in these areas were observed in both structural and functional imaging data of subjects affected by COVID-19 [66]. Interestingly, one study reported a decrease in the volume of the left postcentral gyrus in long COVID-19 patients, which was associated with impairments in executive functions [67]. Furthermore, the same study reported that hyperconnectivity within the right premotor thalamus area was associated with measures of verbal learning, processing speed, and cognitive flexibility, evaluated in subjects 2 years after COVID-19 infection [67]. Another study examining thalamic connectivity with cortical regions found that hypoconnectivity, particularly between the thalamus and motor-related areas, was related to impairments in processing speed [31]. Finally, two studies in COVID-19 cohorts using graph-based measures showed that altered thalamic connectivity strength and network integration were linked to poorer performance in memory and executive functioning [68], as well as in processing speed and cognitive flexibility [69].

Studies in other clinical populations also provided important insights into the associations between CI and thalamo-cortical connectivity. For instance, hyperconnectivity between the thalamus and primary somatosensory cortex was reported in subjects with major depressive disorder in association with worse memory and impairments in processing speed [70]. In subjects with schizophrenia, one study found that hyperconnectivity between the thalamus and bilateral postcentral gyri was associated with impairments in attention and processing speed [71]. Finally, one study focusing on Alzheimer’s disease reported findings similar to ours, showing that hyperconnectivity between the right postcentral gyrus and the left thalamus correlated with CI and deficits in verbal learning [72].

There are inconsistencies across studies, as some reported thalamo-cortical hyperconnectivity, while others observed hypoconnectivity, possibly due to the specific thalamic subregions and cortical area examined, assessment at different post-infection intervals, and the approach used in fMRI data analysis. Despite discrepancies, a growing body of evidence indicates that alterations in thalamo-cortical connectivity play a key role in the pathophysiology of COVID-19-related cognitive dysfunctions.

Emerging findings suggest that alterations in fMRI functional connectivity and the onset of CI may reflect the downstream effects of SARS-CoV-2–related neuroinflammatory and immune processes [15]. Both acute and post-acute phases of the infection are characterized by sustained systemic inflammation and blood–brain barrier (BBB) dysfunctions, which promote microglial reactivity and neuroinflammation within the central nervous system [73]. As also observed in other conditions characterized by CI, persistent immune dysregulation and cytokine signaling can sustain microglial activation over time, potentially leading to chronic alterations in neural efficiency and connectivity [74, 75]. Consistent with this framework, recent evidence shows that patients with long COVID and related CI exhibit elevated inflammatory and cerebrovascular damage-related markers, including interleukin-8, D-dimer, and transforming growth factor-beta (TGF-β) [76–78]. Converging evidence from imaging also seems to support this mechanistic link. For instance, one study using PET in patients with persistent post-COVID cognitive symptoms showed increased microglial activation in the ventral striatum and dorsal putamen, and higher gliosis (an index of inflammatory change) associated with worse processing speed, implicating a possible role of striatal neuroinflammation in CI [79]. Furthermore, a whole-brain rs-fMRI study in COVID-19 patients reported that alterations in DMN and attention network correlated not only with CI, but also with inflammatory markers such as the erythrocyte sedimentation rate and C-reactive protein levels, suggesting a direct coupling between systemic immune activation, FC changes, and cognitive deficits [34]. Increased BBB permeability, reflected by increased levels of glial markers and endothelial activation, has also been investigated as a potential neurobiological mechanism underlying CI [76, 78, 80]. Specifically, the increase in circulating BBB-related proteins (e.g., S100β, Glial fibrillary acidic protein, and Matrix Metalloproteinase-9) indicates loss of neurovascular integrity, which likely enables sustained neuroinflammatory signaling and excitotoxic damage to neurons, ultimately disrupting cerebral networks sustaining cognitive functioning [76, 78, 80]. For instance, a study reported that BBB disruption was directly linked to structural alterations (reduced brain volume and cortical thickness in the superior frontal and middle temporal gyri) in COVID-19 patients with CI, changes that likely translate into functional deficits [76]. Finally, additional pathophysiological phenomena, including hypoxia, endothelial dysfunction, impaired neurogenesis, and microvascular injury, may further exacerbate the vulnerability of fronto-subcortical circuits, particularly in more severe cases of COVID-19 disease [73, 81, 82].

Although the precise causal mechanisms remain to be established, the detection of functional connectivity alterations in long COVID-associated cognitive impairment is consistent with processes such as persistent neuroinflammation, BBB disruption, and reorganization of neural networks following SARS-CoV-2 infection.

We found no significant differences in whole-brain ROI-to-ROI connectivity between COG+ and COG− patients. Prior studies suggest that resting-state FC might be more closely related to cognition in a continuous and dimensional manner, rather than aligning with categorical or diagnostic distinctions, which might explain the lack of group-level differences in our sample [83, 84]. For instance, one study focusing on CI in multiple sclerosis (MS) reported that whole-brain ROI-to-ROI FC clearly differentiated MS patients from healthy controls, yet did not distinguish cognitively impaired from cognitively preserved MS subgroups [84]. Nevertheless, FC values in cerebello-occipital connections correlated strongly with impairments in processing speed within the MS cohort, indicating that FC captured individual variability in cognition despite null group contrasts [84]. Likewise, van Geest et al. found no group differences in the DMN connectivity between MS patients with CI and controls, whereas dynamic DMN connectivity from rest to task explained a substantial proportion of the variance in information-processing speed [83].

The literature on COVID-19 relevant to this topic is highly variable, with some studies reporting increased brain connectivity [62, 85], others showing hypoconnectivity [27], and others reporting no specific alterations of connectivity in subjects with post-COVID-19 CI [35]. Various factors may account for heterogeneity in reported findings: (1) the type of fMRI analysis employed; (2) the size of the study sample; (3) the severity of COVID-19, and (4) the time elapsed between the infection and fMRI data acquisition. We chose a whole-brain approach to examine potential alterations across all brain connections. However, testing over 30,000 connections introduces a substantial multiple-comparisons burden, necessitating stringent significance thresholds that may obscure subtle but biologically meaningful effects [86]. Other studies investigated potential alterations in connectivity among brain regions underlying post-COVID-19 CI, but used different analysis approaches, such as seed-to-voxel analysis [85] or ICA [33, 35]. One study [85] reported increased FC within the visual network and between the thalamus and visual regions in long COVID patients with CI, and another [33] found increased FC in the right middle frontal gyrus alongside decreased FC in the inferior parietal lobule and fronto-parietal junction. Conversely, an outcome in line with our results has been described in one study, where intra- and inter-network FC did not differ between patients with and without CI, while connectivity metrics were related to cognitive performance across the whole sample [35]. Taken together, these findings suggest that subtle disruptions in large-scale cortico-striato and thalamo-cortical loops may manifest as graded changes, without necessarily shifting mean FC values enough to survive stringent multiple-comparison correction in dichotomous COG+/COG− contrasts.

Regarding the sample size, our study included a larger cohort than most previous rs-fMRI studies on post-COVID-19 CI [27, 33, 62, 85]. This factor enhances the reliability of our findings compared to those with smaller sample sizes.

In addition, methodological factors (e.g., severity of respiratory symptoms, time elapsed between infection and fMRI scans, recruitment criteria) might influence study findings, as summarized in a recent review by Nasir et al. [36]. Our study included patients who met specific criteria for disease severity, such as hospitalization for symptomatic infection or a prolonged high fever. However, these criteria might have inadvertently led to the recruitment of subjects who experienced a moderate severity COVID-19 disease. In addition, in our sample, the time elapsed between COVID-19 infection and fMRI acquisition was, on average, 16 months. This interval is among the longest reported in the relevant literature and might have led to the resolution or attenuation of alterations in brain connectivity, making their detection more difficult [27, 29, 30, 34, 87].

The current study presents a few limitations. First, pre-COVID neuroimaging or cognitive data from study participants were not available, thus limiting our ability to infer causal effects of COVID-related neural alterations. Second, the heterogeneity and long time span between the initial onset of COVID-19 and the clinical and MRI assessments of subjects may have added variance to our results. Third, although standardized acquisition protocols across sites were implemented and the recruitment center was used as a covariate, formal harmonization techniques were not applied to FC data. Therefore, residual site-related effects cannot be fully excluded. Finally, because MRI acquisition was approved as an additional component of the study, neuroimaging and cognitive assessments were not conducted concurrently, resulting in a slight delay between the two time points.

In conclusion, our findings indicate that FC alterations within the cortico-striatal and thalamo-cortical circuits may subtend deficits in higher-order executive functions in post-COVID-19 patients and highlight the importance of examining discrete cognitive domains in relation to brain connectivity.

## Supporting information

10.1192/j.eurpsy.2026.12227.sm001 Perrottelli et al. supplementary material Perrottelli et al. supplementary material

## Data Availability

The data that support the findings of this study are available on request from the corresponding authors.

## Supplementary material

The supplementary material for this article can be found at http://doi.org/10.1192/j.eurpsy.2026.12227.

## Long descriptions

### [Figure 1] Long description

The flowchart consists of five main vertical stages in dark blue boxes, with four exclusion branches in light blue boxes to the right.

* Stage 1: Subjects who completed baseline assessment with in-depth cognitive evaluation M C C B n equals 402. An arrow points right to: Subjects who refused to participate to the M R I add-on study n equals 228.

* Stage 2: Subjects with s M R I and r s f M R I data available n equals 174. An arrow points right to: Subjects excluded from the study n equals 27. This includes 21 subjects with a time interval greater than 3 months between M R I acquisition and clinical assessment, and 6 subjects from centers with fewer than 10 participants.

* Stage 3: Eligible subjects n equals 147. An arrow points right to: Subjects excluded after visual inspection of s M R I and r s f M R I data due to artefacts n equals 5.

* Stage 4: Subjects included in the preprocessing pipeline n equals 142. An arrow points right to: Subjects excluded due to excessive head movement and or insufficient number of available slices remaining following preprocessing n equals 6.

* Stage 5: Final study sample n equals 136.

Navigate back to [Figure 1].

### [Table 1] Long description

The table contains four columns: Variable, C O G plus (n equals 52), C O G minus (n equals 84), and t-chi squared-F forward slash p-value.

Demographics:

* Age: C O G plus is 57.30 plus or minus 7.30; C O G minus is 50.73 plus or minus 10.26; p-value is less than 0.001.

* Gender (M forward slash F): C O G plus is 37 forward slash 15; C O G minus is 46 forward slash 38; p-value is 0.057.

* Education (Years): C O G plus is 12.44 plus or minus 3.58; C O G minus is 14.01 plus or minus 3.85; p-value is 0.019.

Clinical Scores:

* M o C A total score: C O G plus is 23.15 plus or minus 2.29; C O G minus is 27.65 plus or minus 1.35; p-value is less than 0.001.

* C O V I D 19 disease severity: C O G plus is 2.14 plus or minus 0.98; C O G minus is 1.89 plus or minus 1.01; p-value is 0.185.

* Time from C O V I D 19 Infection to screening (months): C O G plus is 14.40 plus or minus 8.90; C O G minus is 16.98 plus or minus 8.90; p-value is 0.133.

M C C B Cognitive Domains (C O G plus vs C O G minus):

* Speed of processing: 47.41 plus or minus 11.41 vs 52.08 plus or minus 8.82; p-value is 0.048.

* Attention forward slash vigilance: 44.26 plus or minus 11.32 vs 49.56 plus or minus 10.49; p-value is 0.013.

* Working memory: 42.10 plus or minus 12.78 vs 49.94 plus or minus 8.20; p-value is less than 0.001.

* Verbal learning: 41.49 plus or minus 11.50 vs 50.66 plus or minus 10.70; p-value is less than 0.001.

* Visual learning: 38.90 plus or minus 14.80 vs 52.48 plus or minus 9.73; p-value is less than 0.001.

* Reasoning and problem-solving: 43.69 plus or minus 9.46 vs 51.34 plus or minus 9.39; p-value is less than 0.001.

* Social cognition: 51.27 plus or minus 12.58 vs 53.91 plus or minus 10.03; p-value is 0.126.

* Neurocognitive composite score: 40.28 plus or minus 13.62 vs 51.79 plus or minus 8.46; p-value is less than 0.001.

* Overall composite score: 39.23 plus or minus 12.76 vs 51.10 plus or minus 8.48; p-value is less than 0.001.

Navigate back to [Table 1].

### [Figure 2] Long description

The heatmap is a square grid with both the vertical y-axis and horizontal x-axis labeled with the same sequence of brain regions. From top to bottom on the y-axis and left to right on the x-axis, the regions are Frontal Lobe, Temporal Lobe, Parietal Lobe, Insular Gyrus, Cingulate Gyrus, Occipital Lobe, and Subcortical Nuclei.

A prominent black diagonal line runs from the top-left corner to the bottom-right corner, representing the self-correlation of each R O I. The rest of the matrix is filled with a dense, pixelated pattern of colors representing connectivity values. The distribution appears relatively uniform with no large distinct clusters of high intensity.

To the right of the matrix is a vertical color scale bar. The scale ranges from negative 4.12 at the bottom in dark blue, through 0 in light green at the center, to positive 4.12 at the top in dark orange. The majority of the matrix consists of light green, yellow, and light blue pixels, indicating values near zero or with low positive and negative associations.

Navigate back to [Figure 2].

### [Figure 3] Long description

A semi-transparent 3 D rendering of a human brain viewed from a posterior-superior perspective. Red lines represent functional connectivity pathways between specific R O I s.

At the superior cortical level, four labels are positioned across the top:

* A 6 c d l R and A 6 m R on the right hemisphere.

* A 6 m L and A 6 c d l L on the left hemisphere.

In the medial region, slightly below the top labels, is A 2 4 r v L.

Moving inferiorly toward the subcortical regions, two clusters of labels represent the basal ganglia structures:

* On the right side: G P R, d l P u R, and v m P u R, with a further lateral extension to v I a R.

* On the left side: G P L, d l P u L, and v m P u L.

The red connectivity lines form a dense web-like structure, primarily converging from the superior frontal and precentral areas (A 6 regions) down toward the bilateral putamen and globus pallidus clusters. The lines cross through the medial plane, indicating both ipsilateral and contralateral connections between the cortex and the deeper brain structures.

Navigate back to [Figure 3].

### [Figure 4] Long description

The diagram displays a network of blue lines representing connections within a semi-transparent gray brain shell.

At the central core, a dense cluster of labels represents subcortical and thalamic regions. These include:

* l P F tha L and l P F tha R (Lateral Prefrontal thalamus Left and Right)

* m P F tha L and m P F tha R (Medial Prefrontal thalamus Left and Right)

* r T tha L (Rostral Temporal thalamus Left)

* c T tha L (Caudal Temporal thalamus Left)

* P P tha L (Posterior parietal thalamus Left)

* d Ca L and d Ca R (Dorsal caudate Left and Right)

* N A C R (Nucleus accumbens Right)

Radiating outward from this central hub, blue lines connect to specific cortical regions located in the upper and outer sections of the brain model:

* A 2 L and A 2 R (Postcentral Gyrus Area 2 Left and Right)

* A 7 p c L and A 7 p c R (Superior Parietal Lobule postcentral area 7 Left and Right)

The lines are most concentrated between the thalamic regions and the superior parietal and postcentral areas. Variations in line thickness are due to graphical perspective and the overlapping of multiple connection paths.

Navigate back to [Figure 4].

### [Table 2] Long description

The table consists of four columns: R O I n dot 1, R O I n dot 2, r (correlation coefficient), and p-value.

* Precentral gyrus – caudal dorsolateral area 6 - L is correlated with:

- Dorsolateral putamen – R: r = 0.181, p = 0.038855.

- Dorsolateral putamen – L: r = 0.311, p = 0.000294 (significant).

- Globus pallidus – L: r = 0.295, p = 0.000632 (significant).

* Precentral gyrus – caudal dorsolateral area 6 – R is correlated with:

- Dorsolateral putamen – R: r = 0.191, p = 0.028831.

- Dorsolateral putamen – L: r = 0.284, p = 0.001016 (significant).

- Globus pallidus – L: r = 0.226, p = 0.009588.

- Ventromedial putamen – R: r = 0.212, p = 0.014821.

* Superior frontal gyrus – medial area 6 – L is correlated with:

- Dorsolateral putamen – R: r = 0.197, p = 0.023861.

- Dorsolateral putamen – L: r = 0.233, p = 0.007284.

- Globus pallidus – R: r = 0.189, p = 0.030368.

- Insular gyrus, Ventral agranular insular – R: r = 0.193, p = 0.026907.

- Ventromedial putamen – R: r = 0.199, p = 0.022827.

- Ventromedial putamen – L: r = 0.246, p = 0.004637.

* Superior frontal gyrus – medial area 6 – R is correlated with:

- Cingulate Gyrus, rostroventral area 24: r = 0.182, p = 0.037254.

- Dorsolateral putamen – R: r = 0.197, p = 0.023919.

- Dorsolateral putamen – L: r = 0.288, p = 0.000841 (significant).

- Globus pallidus – R: r = 0.238, p = 0.006144.

- Globus pallidus – L: r = 0.294, p = 0.00064 (significant).

- Insular gyrus, Ventral agranular insular – R: r = 0.214, p = 0.014083.

- Ventromedial putamen – R: r = 0.221, p = 0.011274.

Significant correlations are defined by a p-value threshold adjusted for multiple tests at p less than 0.00135.

Navigate back to [Table 2].

### [Table 3] Long description

The table consists of four columns: R O I n dot 1, R O I n dot 2, r, and p-value.

* For Postcentral gyrus – area 2 - L:

- Caudal temporal thalamus – L: r = -0.210, p = 0.0163.

- Medial prefrontal thalamus – L: r = -0.220, p = 0.0115.

* For Postcentral gyrus – area 2 – R:

- Rostral temporal thalamus – L: r = -0.179, p = 0.0411.

- Caudal temporal thalamus – L: r = -0.271, p = 0.0018.

- Dorsal caudate – L: r = -0.204, p = 0.0197.

- Dorsal caudate – R: r = -0.181, p = 0.0387.

- Lateral prefrontal thalamus – L: r = -0.267, p = 0.0021.

- Lateral prefrontal thalamus – R: r = -0.247, p = 0.0045.

- Medial pre-frontal thalamus – L: r = -0.291, p = 0.000749 (significant at p < 0.00135).

- Medial prefrontal thalamus – R: r = -0.201, p = 0.0213.

- Posterior parietal thalamus – L: r = -0.263, p = 0.0024.

- Rostral temporal thalamus – L: r = -0.200, p = 0.0222.

* For Superior parietal lobule, postcentral area 7 – L:

- Nucleus accumbens – R: r = -0.191, p = 0.0288.

- Rostral temporal thalamus – L: r = -0.180, p = 0.0402.

* For Superior parietal lobule, postcentral area 7 – R:

- Caudal temporal thalamus – L: r = -0.175, p = 0.0450.

- Lateral prefrontal thalamus – L: r = -0.204, p = 0.0192.

- Medial prefrontal thalamus – L: r = -0.200, p = 0.0223.

Navigate back to [Table 3].

### [Figure 5] Long description

A multi-panel figure containing six scatter plots labeled a through f. All plots share a common x-axis titled R P S t-scores ranging from 20 to 70. The y-axis represents Connectivity between specific R O I pairs.

* Panel a. Y-axis is Connectivity between A 6 c d l L and d l P u L. Data points are red with a positive linear regression line. The correlation coefficient r equals 0.311.

* Panel b. Y-axis is Connectivity between A 6 c d l L and G P L. Data points are red with a positive linear regression line. The correlation coefficient r equals 0.295.

* Panel c. Y-axis is Connectivity between A 6 c d l R and d l P u L. Data points are red with a positive linear regression line. The correlation coefficient r equals 0.284.

* Panel d. Y-axis is Connectivity between A 6 m R and d l P u L. Data points are red with a positive linear regression line. The correlation coefficient r equals 0.288.

* Panel e. Y-axis is Connectivity between A 6 m R and G P L. Data points are red with a positive linear regression line. The correlation coefficient r equals 0.294.

* Panel f. Y-axis is Connectivity between A 2 R and m P F t h a L. Data points are blue with a negative linear regression line. The correlation coefficient r equals negative 0.291.

Navigate back to [Figure 5].

### [Table 4] Long description

The table consists of four columns: R O I n dot 1, R O I n dot 2, r, and p-value.

Row 1: Precentral gyrus minus caudal dorsolateral area 6 minus L correlated with Dorsolateral putamen minus L. r equals 0.327, p-value equals 0.000234.

Row 2: Precentral gyrus minus caudal dorsolateral area 6 minus L correlated with Globus pallidus minus L. r equals 0.258, p-value equals 0.00403.

Row 3: Precentral gyrus minus caudal dorsolateral area 6 minus R correlated with Dorsolateral putamen minus L. r equals 0.292, p-value equals 0.00106.

Row 4: Superior frontal gyrus minus medial area 6 minus R correlated with Dorsolateral putamen minus L. r equals 0.259, p-value equals 0.00394.

Row 5: Superior frontal gyrus minus medial area 6 minus R correlated with Globus pallidus minus L. r equals 0.227, p-value equals 0.0117.

Row 6: Postcentral gyrus minus area 2 minus R correlated with Medial pre-frontal thalamus minus L. r equals negative 0.248, p-value equals 0.00582.

Footnote a indicates significant correlations where the p-value threshold was adjusted for multiple tests to p less than 0.0083.

Navigate back to [Table 4].
