# Supplementary material for: Resting-state functional connectivity and cognitive impairment after COVID-19 infection: Evidence from a large-scale fMRI study
Source: Eur Psychiatry. 2026 Jun 8;69(1):e69. doi: 10.1192/j.eurpsy.2026.12227 (PMC13359014; doi:10.1192/j.eurpsy.2026.12227)
Supplement: Perrottelli et al. supplementary material [file S0924933826122275sup001.docx]

**Supplementary material**

**RESTING-STATE FUNCTIONAL CONNECTIVITY AND COGNITIVE IMPAIRMENT AFTER COVID-19 INFECTION: EVIDENCE FROM A LARGE-SCALE fMRI STUDY**

**1. Methods**

*1.1 MRI Scan Protocols*

*1.2 fMRI data preprocessing*

*1.3 Parameters and methodological approach for group-level statistics*

- 1. *Classification based on MCCB results*

**2. Results**

*2.1 Characterization of the fMRI sample and site-related differences*

- 1. *MCCB-based Group Comparison on Resting-State Functional Connectivity*

**3. Figures**

***Figure S1****. Example of the parcellation scheme of the human brain in the BNA, which includes cortical and subcortical ROIs*

1. **Tables**

***Supplementary Table 1.*** *Group comparison of the fMRI study sample of the analysis (n=136) and subjects who only completed the baseline phase of the clinical study (n=266)*

**References**

**1. Methods**

*1.1 MRI Scan Protocols*

MRI scans were acquired with five different 3 Tesla (3T) scanners (Brescia: 3T Siemens Skyra scanner 64-channel head coil; Genoa: 3T Siemens Prisma scanner 64-channel head coil; Naples: MR Discovery 750 General Electric Healthcare, Milwaukee, WI 36-channel parallel head coil; Rome: Philips Achieva 3T Intera 8-channel head coil; Salerno: 3T Siemens Skyra scanner 32-channel parallel head coil). To reduce inter-site variability, MRI data collected across different centers and scanners were harmonized through standardized acquisition protocols. Quality control procedures were systematically applied to identify and exclude datasets affected by motion, artifacts, or inconsistencies in image quality.

Structural T1-weighted images were acquired with high-resolution 3D sequences (3D T1-TFE at Rome; BRAVO at Naples; MPRAGE at Brescia, Genoa and Salerno) with the following parameters: voxel size = 1 × 1 × 1 mm³, matrix = 256 × 256, 192 slices, TR = 2000 ms (7416 ms for the Naples site), TE ≈ 3.06 ms, TI = 850 ms, and flip angle = 8° (9° for the Naples site). rs-fMRI data were obtained using gradient-echo echo-planar imaging (EPI) sequences with the following parameters: TR = 2500 ms (3000 ms for the Rome site), TE = 30 ms, flip angle = 81°, 43 axial slices, slice thickness = 3 mm, spacing = 0 mm, voxel size = 3 × 3 × 3 mm³, and 200 volumes per subject. During rs-fMRI scanning, participants were instructed to rest quietly with their eyes closed, remain still, and stay awake. All sMRI and rs-fMRI data were visually inspected for prominent artifacts (e.g., Gibbs ringing, susceptibility-related distortion), and any scans with detectable defects were excluded from further analysis.

*1.2 fMRI data preprocessing*

Functional data were realigned using SPM realign & unwarp procedure [1], where all scans were coregistered to a reference image (first scan of the first session) using a least squares approach and a 6-parameter (rigid body) transformation, and resampled using b-spline interpolation to correct for motion and magnetic susceptibility interactions. Temporal misalignment between different slices of the functional data (acquired in interleaved bottom-up order) was corrected following SPM slice-timing correction procedure [2], using sinc temporal interpolation to resample each slice BOLD timeseries to a common mid-acquisition time. Potential outlier volumes were identified through the artifact detection tools (ART) [3] whenever the framewise displacement (FD) value was higher than 0.5 mm or the global BOLD signal changed more than 3 standard deviations [4, 5]. A reference BOLD image was computed for each subject by averaging all scans excluding outliers. Participants were excluded from the final sample if, after ART-based scrubbing, fewer than 130 of the 200 volumes remained, or if mean FD exceeded 0.5 mm. Functional and anatomical data were coregistered and normalized into standard MNI space, segmented into grey matter, white matter, and CSF tissue classes, and resampled to 2 mm isotropic voxels following an indirect normalization procedure [5, 6] based on SPM unified segmentation and normalization algorithm [7, 8] with the default IXI-549 tissue probability map template. Last, functional data were smoothed using spatial convolution with a Gaussian kernel of 6 mm full width half maximum (FWHM).

In addition, functional data were denoised using a standard denoising pipeline including the regression of potential confounding effects characterized by white matter timeseries (5 CompCor noise components), CSF timeseries (5 CompCor noise components), motion parameters and their first order derivatives (12 factors), outlier scans (below 108 factors) [4], session effects and their first order derivatives (2 factors), and linear trends (2 factors) within each functional run, followed by bandpass frequency filtering of the BOLD timeseries [9] between 0.0078 Hz and 0.1 Hz. CompCor [10, 11] noise components within white matter and CSF were estimated by computing the average BOLD signal as well as the largest principal components orthogonal to the BOLD average, motion parameters, and outlier scans within subjects’ eroded segmentation masks. From the number of noise terms included in this denoising strategy, the effective degrees of freedom of the BOLD signal after denoising were estimated to range from 30.4 to 80.2 (average 73.4) across all subjects [5].

*1.3 Parameters and methodological approach for group-level statistics*

Connection-level hypotheses were evaluated using multivariate parametric statistics with random-effects across subjects and sample covariance estimation across multiple measurements. Inferences were performed at the level of individual clusters. Cluster-level inferences were based on parametric statistics within and between each pair of networks (Functional Network Connectivity [12]), with networks identified using a complete-linkage hierarchical clustering procedure based on ROI-to-ROI anatomical proximity and functional similarity metrics. Specifically, the hierarchical clustering procedure uses a complete linkage (further distance) method with euclidean distance metric. Distances are computed as a weighted average of differences in connectivity statistics (functional criteria) and differences in spatial location (spatial criteria) between each pair of ROIs (Distances = 0.05*D_anat + 0.95*D_func; D_anat represents the squared euclidean distance between the anatomical 3D centroid coordinates for every pair of ROIs, and D_func is the squared euclidean distance between the connectivity patterns averaged across all subjects for every pair of ROIs) [13].

*1.4 Classification based on MCCB results*

As an additional analysis, participants were classified as cognitively impaired (COG+) or non-impaired (COG−) based on their performance on the MCCB at baseline. COG+ subjects were defined relative to the Italian normative sample as exhibiting at least mild deficits in two or more cognitive domains, with mild impairment defined as scores 1–2 standard deviations below the normative mean.**2. Results**

*2.1 Characterization of the fMRI sample and site-related differences*

We examined whether the current fMRI subsample differed from the baseline-phase participants of the clinical study [14] who were not included in this analysis in terms of demographic characteristics, COVID-19 severity, and cognitive profiles, using independent-samples t-tests, chi-square tests, and a Multivariate Analysis of Variance (MANOVA). No significant differences were observed between subjects included in the current fMRI study (n=136) and those who participated in the baseline clinical study (n=266) in age, education, male/female ratio, COG+/COG- ratio (p>0.05) and MCCB scores [Wilks’ Λ = 0.962, F_(9, 335)_ = 1.461, p = 0.161] (**Supplementary Table 1**). However, subjects included in the present fMRI study showed lower severity of COVID-19 disease and a longer time between the onset of COVID-19 symptoms and the study recruitment, as compared to the entire group of subjects included in the clinical-neurocognitive study (**Supplementary Table 1**). Furthermore, we investigated if severity of CI varied depending on whether subjects were hospitalized (n=81) or not (n=55). A significantly lower MoCA total score (t_(134)_ = 2.710, p = 0.008) was recorded in subjects who were hospitalized (Mean ± S.D. = 26.7 ± 2.23), as compared to non-hospitalized patients (Mean ± S.D. = 25.4 ± 3.05), suggesting that hospitalization and worse COVID-19 disease pathology were risk factors for developing CI, as it shown by the clinical-neurocognitive study [14]. No significant difference was observed between the two groups on the average of the MCCB neurocognitive composite score (t_(134)_ = 1.129, p = 0.261).

Finally, we examined whether the time between COVID-19 infection and screening differed across recruitment sites. Among the final 136 subjects included in the analyses, 90 were recruited from the University of Brescia (Brescia center), 28 from the University of Campania “Luigi Vanvitelli” (Naples center), and 18 from the University of Salerno (Salerno center). A Kruskal–Wallis H test showed a significant result indicating differences in recruitment timing among the three centers [H_(2)_ = 73.77, p < 0.001]. Post hoc pairwise comparisons with Bonferroni correction revealed that participants recruited from the Brescia center had a significantly longer time between infection and screening than those from Naples (p < 0.001) and Salerno (p < 0.001). There was no significant difference between subjects recruited from Naples and Salerno centers (p > 0.05).

*2.2 MCCB-based Group Comparison on Resting-State Functional Connectivity*

As an additional analysis, the second-level ROI-to-ROI analyses, performed across the 246 BNA ROIs, have also been performed based on the MCCB classification. Specifically, based on the MCCB results, the COG+ group included 50 subjects (36.8%), and the COG- group 86 (63.2%). Group-level contrasts (COG+ > COG- and COG- > COG+) were then tested within a GLM framework. No significant between-group differences in FC emerged, either at the individual connection level or when applying cluster-level correction. Controlling for age, sex, years of education, time between COVID-19 infection and screening, and recruitment site (entered individually and in combination as covariates in the GLM) did not modify the results.

**3. Figures**

**Figure S1.** *Example of the parcellation scheme of the human brain in the BNA, which includes cortical and subcortical ROIs.*

**
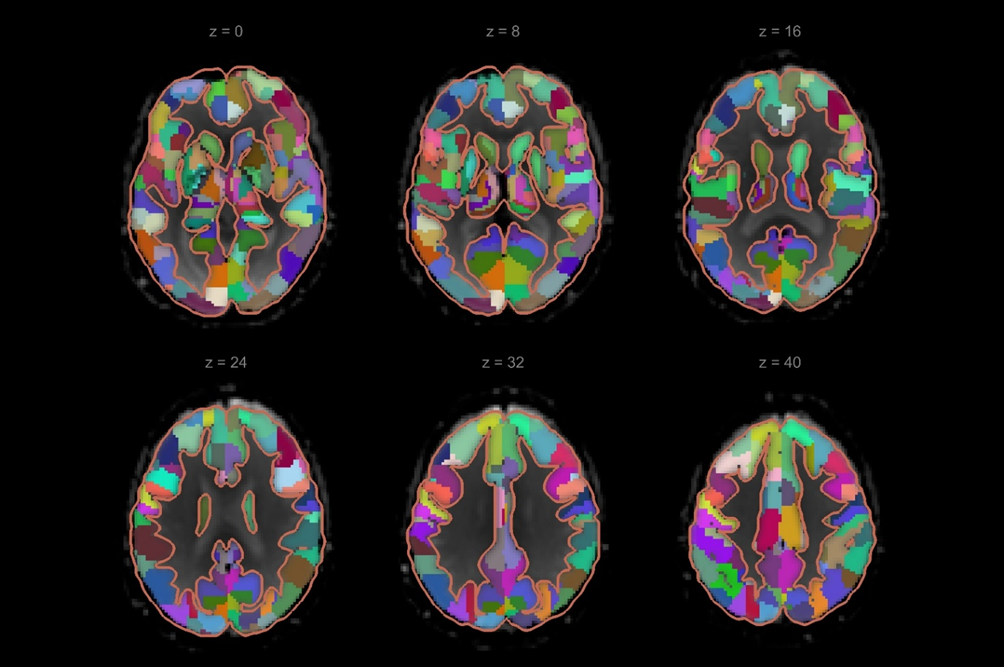
**

**4. Tables**

**Supplementary Table 1.** Group comparison between the fMRI study sample included in the analysis (n=136) and subjects who only completed the baseline phase of the clinical study (n=266)

|  | **fMRI sample**  **(n=136)** | **Clinical sample (n=266)** | **t-χ^2^-F / p- value** |
| --- | --- | --- | --- |
| Age ^a^ | 53.24 ± 9.75 | 52.50 ± 10.15 | 0.705/0.481 |
| Gender (M/F) ^a^ | 83/53 | 165/101 | 0.063/0.802 |
| Education (Years) ^a^ | 13.41 ± 3.80 | 13.36 ± 3.73 | 0.199/0.906 |
| MoCA Total Score ^b^ | 25.93 ± 2.81 | 25.40 ± 2.77 | 1.836/0.067 |
| COG+ /COG- within the sample (% of COG+) ^b^ | 52/84 (38.2%) | 126/140 (47.3%) | 3.042/0.081 |
| COVID-19 disease severity ^b^  (at the time of symptomatic infection) | 1.99 ± 1.00 | 2.26 ± 0.99 | 2.363/0.019 |
| Time from COVID-19 Infection  to screening (Months) ^b^ | 16.00 ± 8.96 | 10.71 ± 7.76 | **5.781/<0.001*** |
| MCCB Speed of processing ^c^ | 50.26 ± 10.13 | 48.83 ± 9.56 | 1.373/0.171 |
| MCCB Attention/Vigilance ^c^ | 47.50 ± 11.08 | 48.34 ± 10.75 | 0.675/0.500 |
| MCCB Working memory ^c^ | 46.89 ± 10.88 | 45.17 ± 10.33 | 1.531/0.126 |
| MCCB Verbal Learning ^c^ | 47.09 ± 11.86 | 44.84 ± 11.02 | 1.873/0.062 |
| MCCB Visual Learning ^c^ | 47.19 ± 13.63 | 44.63 ± 14.63 | 1.677/0.094 |
| MCCB Reasoning and Problem Solving ^c^ | 48.36 ± 10.10 | 46.51 ± 10.88 | 1.634/0.103 |
| MCCB Social Cognition ^c^ | 52.89 ± 11.12 | 49.77 ± 11.54 | 2.547/0.011 |
| MCCB Neurocognitive Composite Score ^c^ | 47.33 ± 12.09 | 44.80 ± 10.62 | 1.983/0.048 |
| MCCB Overall Composite Score ^c^ | 46.50 ± 11.82 | 44.47 ± 10.97 | 1.578/0.058 |

COG+: subjects who presented a MoCA score<26; COG-: subjects who scored ≥ 26 on the MoCA evaluation.

* significant difference (*p-value threshold adjusted for multiple tests)*: *a) p<0.0125 for demographic variables;* *b) p< 0.0125 for MoCA score & COVID-19 disease variables; c) p<0.0055 for MCCB variables*

**References**

[1] Andersson JL, Hutton C, Ashburner J, Turner R, Friston K. Modeling geometric deformations in EPI time series. Neuroimage. 2001;13(5):903-19. https://doi.org/10.1006/nimg.2001.0746.

[2] Sladky R, Friston KJ, Tröstl J, Cunnington R, Moser E, Windischberger C. Slice-timing effects and their correction in functional MRI. Neuroimage. 2011;58(2):588-94. https://doi.org/10.1016/j.neuroimage.2011.06.078.

[3] Whitfield-Gabrieli S, Nieto-Castanon A, Ghosh S. Artifact detection tools (ART). In: Release Version, 7(19), 11 ed.: Cambridge, MA; 2011.

[4] Power JD, Mitra A, Laumann TO, Snyder AZ, Schlaggar BL, Petersen SE. Methods to detect, characterize, and remove motion artifact in resting state fMRI. Neuroimage. 2014;84:320-41. https://doi.org/10.1016/j.neuroimage.2013.08.048.

[5] Nieto-Castanon A. Preparing fMRI Data for Statistical Analysis*.* 2022. https://doi.org/10.48550/arXiv.2210.13564.

[6] Calhoun VD, Wager TD, Krishnan A, Rosch KS, Seymour KE, Nebel MB, et al. The impact of T1 versus EPI spatial normalization templates for fMRI data analyses. Hum Brain Mapp. 2017;38(11):5331-42. https://doi.org/10.1002/hbm.23737.

[7] Ashburner J, Friston KJ. Unified segmentation. Neuroimage. 2005;26(3):839-51. https://doi.org/10.1016/j.neuroimage.2005.02.018.

[8] Ashburner J. A fast diffeomorphic image registration algorithm. Neuroimage. 2007;38(1):95-113. https://doi.org/10.1016/j.neuroimage.2007.07.007.

[9] Hallquist MN, Hwang K, Luna B. The nuisance of nuisance regression: spectral misspecification in a common approach to resting-state fMRI preprocessing reintroduces noise and obscures functional connectivity. Neuroimage. 2013;82:208-25. https://doi.org/10.1016/j.neuroimage.2013.05.116.

[10] Behzadi Y, Restom K, Liau J, Liu TT. A component based noise correction method (CompCor) for BOLD and perfusion based fMRI. Neuroimage. 2007;37(1):90-101. https://doi.org/10.1016/j.neuroimage.2007.04.042.

[11] Chai XJ, Castañón AN, Ongür D, Whitfield-Gabrieli S. Anticorrelations in resting state networks without global signal regression. Neuroimage. 2012;59(2):1420-8. https://doi.org/10.1016/j.neuroimage.2011.08.048.

[12] Jafri MJ, Pearlson GD, Stevens M, Calhoun VD. A method for functional network connectivity among spatially independent resting-state components in schizophrenia. Neuroimage. 2008;39(4):1666-81. https://doi.org/10.1016/j.neuroimage.2007.11.001.

[13] Nieto-Castanon A. Handbook of functional connectivity Magnetic Resonance Imaging methods in CONN*.* 2020. https://doi.org/10.56441/hilbertpress.2207.6598.

[14] Galderisi S, Perrottelli A, Giuliani L, Pisaturo MA, Monteleone P, Pagliano P, et al. Cognitive impairment after recovery from COVID-19: Frequency, profile, and relationships with clinical and laboratory indices. Eur Neuropsychopharmacol. 2024;79:22-31. https://doi.org/10.1016/j.euroneuro.2023.11.001.
